# Squamous Papilloma on the Hard Palate: A Rare Clinical Entity

**DOI:** 10.7759/cureus.38710

**Published:** 2023-05-08

**Authors:** Karthik Rajaram Mohan, Saramma Mathew Fenn, Ravikumar Pethagounder Thangavelu

**Affiliations:** 1 Oral Medicine, Vinayaka Mission's Sankarachariyar Dental College, Vinayaka Mission's Research Foundation, Salem, IND; 2 Oral Medicine and Radiology, Vinayaka Mission's Sankarachariyar Dental College, Vinayaka Mission's Research Foundation, Salem, IND

**Keywords:** verruca vulgaris, cowden syndrome, excision biopsy, human papillomavirus, squamous papilloma

## Abstract

Squamous papilloma is a benign, exophytic, soft tissue tumour caused by the rapid proliferation of stratified squamous epithelium. It typically manifests in the oral cavity as a painless, soft, non-tender, pedunculated growth that resembles a cauliflower. This case report of squamous papilloma on the hard palate sheds light on the etiopathogenesis, types, clinical features, differential diagnosis, and management modalities.

## Introduction

Squamous papilloma is a benign soft tissue tumour caused by the proliferation of the stratified squamous epithelium, resulting in papillary or verruciform mass. Squamous papilloma is usually asymptomatic and often diagnosed between 10 and 50 years of age incidentally. Although literature reviews stated its common occurrence on the soft palate, uvula, and tongue [1]. Papilloma is a benign soft tissue tumour derived from epithelium on a vascular connective tissue resembling a nipple-like protuberance of a mammary gland. Papilloma is derived from a modern Latin hybrid word "papilla", meaning "nipple", the English word "papula", meaning swelling or pimple, and the Greek word "oma", meaning "tumour". Papilloma is a non-enveloped double-stranded DNA virus. This case reports the occurrence of squamous papilloma on the hard palate. Squamous papilloma clinically appears as a sessile, pedunculated mass, which is usually asymptomatic, cauliflower-like surface, appearing either the same or reddish in colour as the adjacent mucosa, and is non-tender on palpation. The various types of papillomas include fungiform papillomas (Ringertz tumour), inverted Schneiderian papillomas, cylindrical cell papillomas, multifocal papillomas, and juvenile-onset laryngeal papillomas. Papillomas are classified based on the site of anatomical location. Squamous papilloma and oral florid papillomatosis occur in the oral mucous membrane, urothelial papillomas occur in the transitional epithelium lining the urinary bladder, intraductal papillomas are those that occur in the breast, oesophageal papillomas are those that occur in the oesophagus, papillomas that occur in bronchus are called bronchial papillomas, and papillomas that occur in the choroid plexus of the brain are called choroidal papillomas. Conjunctival papillomas are those that occur in the eye near the lacrimal punctum caused by human papillomavirus (HPV) 6, 11, 16, 33, 34, and 35. Cutaneous papillomas are those that occur on the skin. Anogenital papillomas (venereal warts) are caused by HPV 2, 3, 6, 11, 16, 18, 30, 31, and 32.

Fungiform papillomas are broad-based masses that can be pink or tan in colour and include papillary or warty surface projections and usually develop close to the nasal septum and consist of unilateral obstruction and epistaxis. HPV 6, 11, 16, and 18 and the Epstein-Barr virus are the two viruses that produce papillomas that are clinically seen as pink or tan, with polypoid nodular growth that primarily emerges from the lateral nasal cavity wall or a paranasal sinus, generally the antrum.

Cylindrical cell papillomas in the nasal cavity or maxillary antrum typically present as a beefy-reddish brown mass with a multinodular surface. Clinically, several scattered soft, non-tender, flattened, or rounded papules generally are clustered and have the same colour as normal mucosa, known as multifocal papillomas caused by HPV 13 and 32. In addition, juvenile-onset HPV 6 and 11 induce laryngeal papillomatosis, which develops in the larynx during childhood and causes sudden hoarseness in children.

Papillomas are also classified as solitary papillomas, such as squamous papilloma, conjunctival papilloma, intraductal papillomas, and endobronchial papillomas. Multiple papillomas include laryngeal papillomatosis, Cowden syndrome, and WHIM (warts, hypogammaglobulinemia, infections, and myelokathexis) syndrome. Recurrent papillomas are papillomas with a recurrent nature such as laryngeal papillomas.

Risk factors for HPV include sexual transmission from infected partners, immunosuppressive states like HIV, illicit drug abuse, previous history of genital chlamydial infection, gonorrhoea, genital herpes, or trichomoniasis, and co-infection with other sexually transmitted diseases, such as *Chlamydia trachomatis* and herpes simplex virus type 2.

## Case presentation

**Figure 1:**
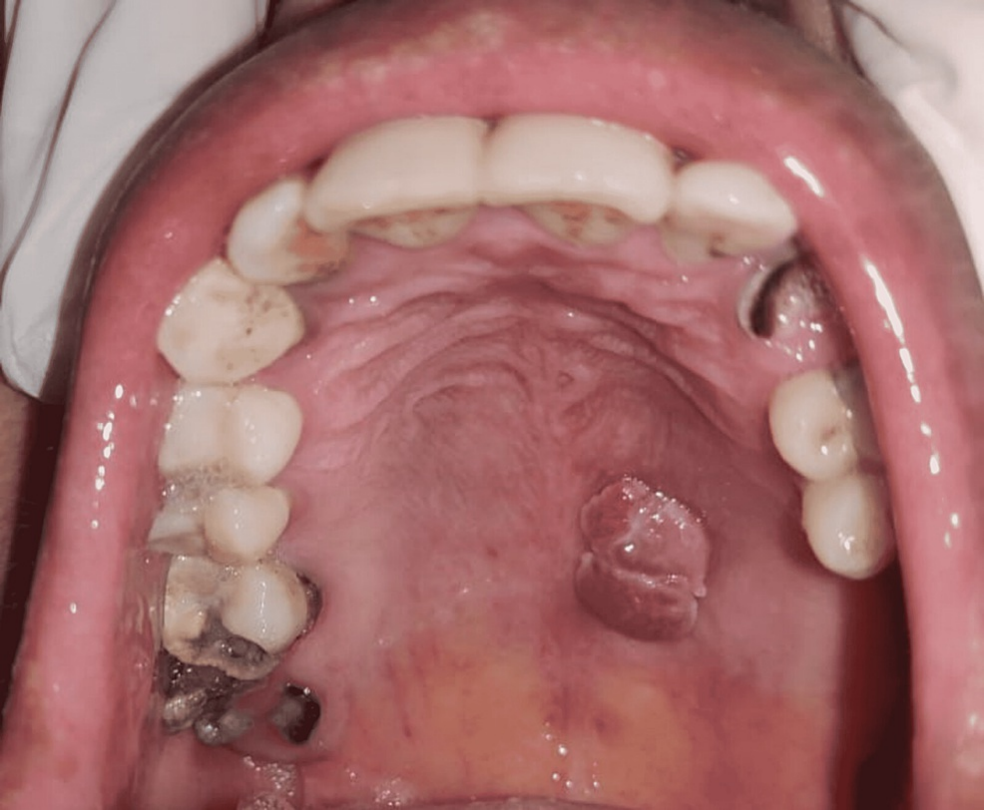
A pedunculated cauliflower-like growth on the left lateral portion of the hard palate 1 mm away from the mid-palatal raphe

A 27-year-old female reported a chief complaint of growth on the hard palate for the past six months. On eliciting the history, she stated that it started as a small growth around nine months back and gradually increased in size and attained the present size of 1.5 × 2 cm (Figure 1).

**Figure 2:**
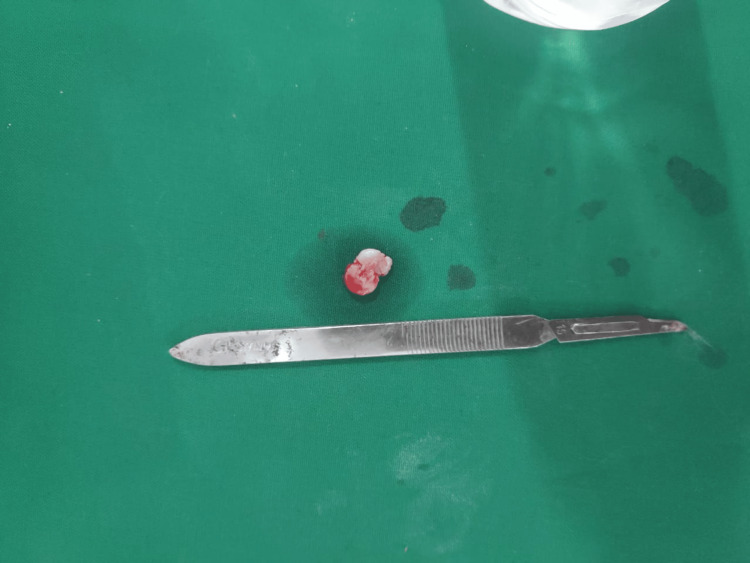
Excised growth specimen

On inspection, a growth measuring about 1.5 x 2 cm was present on the left lateral portion of the hard palate. The growth extended anteriorly 5 cm away from the incisive papilla, medially 0.5 cm away from the mid-palatal raphe, and posteriorly 2 cm away from the fovea palatinae. It was cauliflower-like in shape and the surface appeared more reddish near the superior aspect of the growth. All the findings regarding the site, size, shape, surface, and extent were confirmed on palpation. The growth was firm in consistency and non-tender on palpation. There was no history of rapid growth, paresthesia, or numbness associated with the growth ruling out the possibility of malignancy. A thorough clinical examination revealed an absence of similar growth elsewhere in her body. Family history was not significant. Her medical history revealed seropositivity for HPV subtype 6. There was no harmful habit of smoking or tobacco chewing, alcohol, and intravenous drug abuse. A provisional diagnosis of papilloma was made based on the clinical findings of the presence of an exophytic, sessile, cauliflower-like growth. An excision biopsy of the growth was done under local anaesthesia (Figure 2).

**Figure 3:**
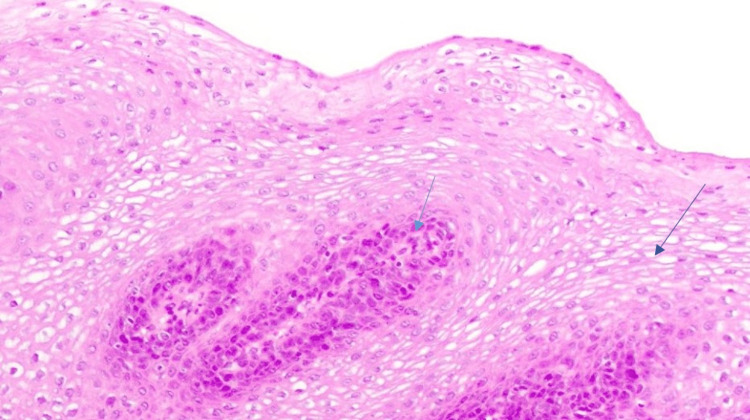
Histopathological examination Hematoxylin and eosin stain (40x) revealed hyperplastic squamous epithelial cells and koilocytosis (squamous epithelial cells with coarse thick chromatin arranged in a perinuclear pattern).

The histopathological examination (40x) revealed koilocytosis (squamous epithelial cells that exhibit thick coarse chromatin) and hyperplastic squamous epithelium (Figure 3).

## Discussion

Papilloma is defined as "a benign tumour of the hyperplastic squamous epithelium occurring clinically as an exophytic, painless, asymptomatic, soft tissue mass with a cauliflower-like surface" caused by the HPV [2]. Papilloma is a non-enveloped, double-stranded DNA virus. More than 200 subtypes of papilloma have been identified. HPV 16 and HPV 18 subtypes have a high propensity for cervical cancer. The p53 protein is induced to degrade by the E6 oncoprotein that is encoded by HPV types 16 and 18. Papilloma can occur anywhere in the body and is named according to the site of location in the human body. The various types of papillomas and their site of location are described in Table 1.

**Table 1:** Types of papillomas and their location in the human body

Types of papillomas	Site of location
Squamous papilloma	Oral mucous membrane
Choroidal plexus papillomas	Brain
Urothelial papillomas	Urinary tract
Conjunctival papillomas	Lacrimal punctum near the lacrimal gland
Laryngeal papillomas	Larynx
Intraductal papillomas	Breast
Cutaneous papillomas	Skin
Tracheal papillomatosis	Trachea
Bronchial papillomas	Bronchus
Oesophageal papillomas	Oesophagus
Anogenital papillomas	Anogenital region, cervix, vulva, and vagina

Papillomas are classified according to their occurrence as either isolated/solitary, multiple, or recurrent (Table 2).

**Table 2:** Classification of papillomas based on the type of occurrence

Solitary/isolated	Squamous papillomas, conjunctival papillomas, endobronchial papillomas, intraductal papillomas
Multiple	Laryngeal papillomas, Cowden syndrome, WHIM (warts, hypogammaglobulinemia, infections, and myelokathexis) syndrome
Recurrent	Laryngeal papillomas

Based on the potential for risk of infections, HPV is classified as high-risk and low-risk HPV (Table 3).

**Table 3:** Classification of HPV based on the risk of infection HPV: human papillomavirus.

High-risk HPV	HPV 16, 18, 31, 33, 45, 52, 58
Low-risk HPV	HPV 6, 11

The most common sites of predilection for papillomas include the soft palate, buccal mucosa, and vermilion border of lips, tongue, and uvula [3,4].

Etiopathogenesis of HPV

Entry of HPV Into Susceptible Host

Epithelial cells are primarily infected by papillomavirus because of the epithelial tropism. The incoming virion of papillomavirus interacts with extracellular heparin sulphate proteoglycans on the basement membrane of the oral squamous epithelium following a micro-abrasion.

Nucleocapsid Uncoating and Disassembly

The L1 and L2 capsid proteins of papillomavirus undergo conformational modifications as a result of this contact, enabling the virion to be delivered into the host cell following which L2 cleavage takes place by furin, and the virion is internalised through a process related to micropinocytosis. The viral DNA is thought to remain linked to L2 during this process. Until the start of mitosis, the L2-DNA complex engages to the trans-Golgi network and stays there. The trans-Golgi network naturally vesiculates during mitosis, and the vesicle-bound viral DNA enters the nucleus. By metaphase, the viral DNA has entered the chromosomes of the host. The viral DNA can be detected linked to nuclear ND10 bodies after mitosis. The capsid proteins L1 and L2 are encoded in the late region. Between the L1 and E6 open reading frames (ORFs), the upstream regulatory region (URR) or locus control region (LCR) houses the viral replication origin as well as binding sites for cellular and viral transcription factors. There are three main phases of replication in the viral replication cycle.

Amplification

The viral E1 and E2 replication proteins support initial, confined viral DNA amplification. The viral E1 helicase is recruited when the viral E2 protein binds to its binding sites in the viral origin of replication, enabling viral replication. The viral genome is kept at a relatively low but constant copy number in the proliferating cells of a clonally enlarged population of infected cells during maintenance replication, which follows this first burst of replication.

Differentiation

Finally, differentiation-dependent genome amplification and the final production of offspring virions occur as an infected cell completes cellular differentiation. The papillomavirus creates an S-phase-like condition in differentiated cells during maintenance replication. The viral E6 and E7 proteins hijack the cellular environment through a variety of protein-protein interactions, enabling viral reproduction in differentiated cells. The viral life cycle's maintenance phase might persist for months or years. The viral E2 protein controls replication in addition to playing a crucial function in maintenance by ensuring that the viral genomes are accurately distributed into the daughter cells. The viral DNA has been amplified to a high copy number in the upper layers of differentiated epithelia. The cellular DNA damage response is necessary for the vegetative stage of the viral life cycle. To form particles encasing the viral DNA, the viral capsid proteins self-assemble. Infectious virions are released as the cells slough off into the environment like bio-fluids such as saliva and amniotic fluid concluding the viral life cycle.

Transmission of HPV

The transmission of HPV occurs vertically from mother to child through amniotic fluid during birth through vaginal delivery. Transmission among adults can occur through infected partners with underlying HIV. The HPV exhibits epithelial tropism and has a high affinity for tonsillar crypts and squamous epithelial lining of the oral mucous membrane. The HPV can be shed in saliva due to shedding by desquamation of squamous epithelial cells. Transmission of HPV also occurs by autoinoculation to the oral cavity by saliva. The HPV has a longer incubation period that varies from three to 10 months.

This case report presents a unique case of squamous papilloma on the hard palate. The differential diagnoses include verruca vulgaris (common warts), verruciform xanthoma, inflammatory papillary hyperplasia of the palate, Cowden syndrome (cardiofaciocutaneous syndrome), Gorlin-Goltz syndrome, and WHIM syndrome. The various differential diagnoses for papillomas are presented in Table 4.

**Table 4:** Differential diagnoses for papillomas HPV: human papillomavirus.

Lesion	Aetiology	Clinical appearance
Verruca vulgaris (common warts)	HPV 2, 4, 6, 40	Clinically oral lesions appear always as white projections papule or nodule and are pedunculated. Can reach a size of <5 mm. Multiple lesions are common. Occasionally keratin accumulates on its surface, which results in a hard surface projection called cutaneous or keratin horn
Condyloma acuminatum (venereal warts)	HPV 2, 6, 11, 53, 54	Pink, sessile, exophytic, non-tender, blunted surface projections with an average size of 1 to 1.5 cm in diameter. Can grow as large as 3 cm
Verruciform xanthoma	Candidiasis, T cell-mediated immunological disorders, skin disorders like acanthosis nigricans	The biphasic appearance of white or yellow blunted projections is due to the accumulation of lipid-laden macrophages (foam cells). Immunopositivity for cathepsin B and CD68 (KP1)
Inflammatory papillary hyperplasia of the palate	Chronic irritation from ill-fitting dentures, poor oral hygiene	Papillary projections beneath ill-fitting dentures
Acrochordon (skin tags)	Chronic friction between skin folds, ageing, obesity	Solitary, finger-like papillary projection on the surface of the skin
Oral florid papillomatosis	Tobacco chewing	A type of verrucous carcinoma that occurs on the tongue, larynx, oesophagus, and maxillary antrum and resembles papilloma with multiple papillary projections on its surface
Cowden syndrome (cardiofaciocutaneous syndrome)	Mutation in HRAS gene	Short stature delayed development and intellectual disability, arrhythmia, septal defects, hypertrophic cardiomyopathy, cutaneous papillomas, muscular hypotonia, and increased incidence of rhabdomyosarcoma
Gorlin-Goltz syndrome	Mutation or deletion in PORCN *(Xp11.23)*	Papillomas occur in the oesophagus and foot (pedal papillomas)
WHIM (warts, hypogammaglobulinemia, infections, and myelokathexis) syndrome	7 transmembrane protein chemokine receptor CXCR4 defect in stem cells/progenitor cells	Warts, hypogammaglobulinemia, infections, retention of mature neutrophils (myelokathexis)

Laryngeal papillomas occurring in the larynx can obstruct airways. Inflammatory palatal papillomatosis also occurs on the tissue-bearing surface of the hard palate among constant complete denture wearers who do not maintain or remove the dentures, even during sleeping at night. Verruca vulgaris, also called common warts, is seen among children. Verruca vulgaris occurs as pedunculated soft tissue mass with verruciform white finger-like projections on the plantar surfaces of the fingers and is commonly transmitted as a result of autoinoculation of HPV 2 and 4 by contact with saliva. Condyloma acuminatum (condyloma is a Greek word meaning round and acuminatum is a Latin word meaning to become pointed) is characterised by whitish or pinkish nodules on the hard palate, tongue, and floor of the mouth and appears as fleshy papules more commonly located in the anogenital region [5]. The various literature reviews on squamous papilloma are described in Table 5.

**Table 5:** Literature reviews on squamous papillomas

Author	Year	Clinical description
Ramanathan et al. [1]	2021	Squamous papilloma on the hard palate is a clinical rarity
Jaya et al. [2]	2020	Squamous papilloma is characterised by painless growth
Stojanov [3]	2020	Excisional biopsy of squamous papilloma is curative. Has rare recurrences
Chaitanya et al. [4]	2018	The common sites of occurrence of oral papillomas include the tongue, soft palate, and uvula
Babaji et al. [6]	2014	Papillomas appear like cauliflower-like painless soft tissue mass
Al-Khateeb [7]	2009	22 cases (5%) among 883 benign soft-tissue masses are seen among the female population in the northern Jordanian population

Cipriani et al. observed the development of HPV-related squamous cell carcinoma in two siblings with WHIM syndrome, caused by a change in the 7-transmembrane protein chemokine receptor CXCR4, which is found in a number of stem cells and progenitor cells but whose function is not fully understood. Warts, hypogammaglobulinemia, infections, and retention of mature neutrophils in the bone marrow (myelokathexis) are all symptoms of the uncommon autosomal dominant condition known as WHIM. It has also been linked to an increased risk of HPV infections [8]. Buschke-Lowenstein tumours are giant-sized condyloma acuminatum caused by HPV 16 and 18 subtypes and can have a psychological effect of anxiety and fear [9]. The various surgical treatment modalities for papilloma are presented in Table 6. The various medical management options for papilloma are presented in Table 7.

**Table 6:** Surgical treatment modalities for papilloma

Surgical treatment modalities	Advantages	Disadvantages
Surgical excision biopsy	Can be sent for histopathological examination	Cannot be done for large-sized papillomas. Inaccessible in anatomical areas such as the larynx and oesophagus. Prolonged healing period
Cryotherapy	Heals rapid by the thawing of tissues	Needs specialized cryoprobes that use liquid nitrogen
Electrocautery	Less recurrence rate	Can cause burnout of the tissues at the surgical site. Histopathological examination cannot be done after this procedure as it causes burnout of tissues
Laser-assisted excision; Co2 laser and potassium titanyl phosphate (KTP) laser	Bloodless, faster healing time, no scar tissue formation after laser excision, KTP laser has 532 nm increased durability, intense pulse for effective removal of respiratory papillomatosis involving larynx (vocal cords)	Requires preactivation of the diode laser tip before excision of tissue
Radiofrequency ablation (RFA)	Rare recurrence rate	Need for specialized equipment
Photodynamic therapy (PDT)	Minimal scar formation	Requires injection of photosensitizer drug - aminolevulinic acid. Requires specialised photo-intensifying unit

**Table 7:** Medical management of papilloma

Medication	Dosage
10-25% podophyllin resin	Weekly for a duration of up to 4 weeks
5% imiquimod cream	3 times per week for ~16 weeks
Podofilox (0.5% solution or gel)	Twice daily for 3 days followed by 4 days without therapy. This cycle is repeated four times
Methotrexate (intralesional)	2 mg/ml for treatment of plantar warts
Bevacizumab (sublesional)	12.5 mg for recurrent respiratory papillomatosis
Cidofovir (sublesional)	2-57 mg every 4-8 weeks for respiratory papillomatosis
Interferon-α injections	3 million IU; 3 times per week for 3 weeks
Cantharidine solution	0.7% for skin warts

Prevention of HPV infections

The HPV vaccine, which is suggested between the ages of 9 and 24, helps to prevent papillomavirus infections. HPV vaccine administration does not completely confer protection against or prevent HPV infections (Table 8) [10].

**Table 8:** HPV vaccines HPV: human papillomavirus.

Name of the vaccine	HPV subtype	Type of vaccine	Dosage and administration
Cervarix	HPV 16-18	Bivalent vaccine expression medium: Trichoplusia ni insect cell line infected with L1 encoding recombinant baculovirus. Adjuvant: 50 µg monophosphoryl lipid A (AS04) absorbed on 500 µg aluminium hydroxide	0.5 mL/dose. First and second dosage at a one-month interval. Third dosage at 6-month interval intramuscularly - deltoid upper forearm
Gardasil 9	HPV 6, 11, 16, 18	Nonavalent vaccine expression medium: *Saccharomyces cerevisiae* (Baker's yeast) expressing L1. Adjuvant: 500 úg aluminium hydroxyphosphate sulphate	0.5 mL/dose. First, second, and third dosages at 0, 2, and 6 months intervals

## Conclusions

Squamous papilloma is a benign soft tissue cauliflower-like growth caused by the hyperplastic proliferation of squamous epithelium induced by HPV 6 and 11. Papillomas are incidentally observed during a clinical examination. Squamous papillomas can appear clinically as sessile, exophytic growth on the hard palate. Treatment of such squamous papillomas must not be neglected. A detailed history and clinical examination are necessary, and the patient must be educated about the excision of such lesions. The recurrence rate of such papilloma is rare, except in immunocompromised patients with acquired immunodeficiency syndrome (AIDS).
